# Longitudinal Changes in Diffusion Tensor Imaging Following Mild Traumatic Brain Injury and Correlation With Outcome

**DOI:** 10.3389/fncir.2019.00028

**Published:** 2019-05-07

**Authors:** Bo Yin, Dan-Dong Li, Huan Huang, Cheng-Hui Gu, Guang-Hui Bai, Liu-Xun Hu, Jin-Fei Zhuang, Ming Zhang

**Affiliations:** ^1^Department of Medical Imaging, The First Affiliated Hospital of Xi’an Jiaotong University, Xi’an, China; ^2^Department of Neurosurgery, The Second Affiliated Hospital and Yuying Children’s Hospital of Wenzhou Medical University, Wenzhou, China; ^3^Department of Radiology, The Second Affiliated Hospital and Yuying Children’s Hospital of Wenzhou Medical University, Wenzhou, China; ^4^Department of Rehabilitation Medicine, The Second Affiliated Hospital and Yuying Children’s Hospital of Wenzhou Medical University, Wenzhou, China

**Keywords:** mild traumatic brain injury, diffusion tensor imaging, longitudinal changes, fractional anisotropy, neuropsychological test

## Abstract

The chronic consequences of traumatic brain injury (TBI) may contribute to the increased risk for early cognitive decline and dementia, primarily due to diffusion axonal injury. Previous studies in mild TBI (mTBI) have been controversial in describing the white matter tract integrity changes occurring at acute and subacute post-injury. In this prospective longitudinal study, we aim to investigate the longitudinal changes of white matter (WM) using diffusion tensor imaging (DTI) and their correlations with neuropsychological tests. Thirty-three patients with subacute mTBI and 31 matched healthy controls were studied with an extensive imaging and clinical battery. Neuroimaging was obtained within 7 days post-injury for acute scans and repeated at 1 and 3 months post-injury. Using a region-of-interest-based approach, tract-based spatial statistics was used to conduct voxel-wise analysis on diffusion changes in mTBI and was compared to those of healthy matched controls, scanned during the same time period and rescanned with an interval similar to that of patients. We found decreased fractional anisotropy (FA) values in the left anterior limb of internal capsule (ALIC) and right inferior fronto-occipital fasciculus (IFOF) during the 7 days post-injury, which showed longitudinal evidence of recovery following 1 month post-injury. Increased FA values in these two tracts at 1 month post-injury were positively associated with better performance on cognitive information processing speed at initial assessment. By contrast, there were also some tracts (right anterior corona radiata, forceps major, and body of corpus callosum) exhibiting the continuing loss of integrity sustaining even beyond 3 months, which can predict the persisting post-concussion syndromes. Continuing loss of structural integrity in some tracts may contribute to the persistent post-concussion syndromes in mTBI patients, suggesting certain tracts providing an objective biomarker for tracking the pathological recovery process following mTBI.

## Introduction

The United States Centers for Disease Control and Prevention estimates that mild traumatic brain injury (TBI) is experienced in 70–90% of TBI-related emergency department (ED) visits (Taylor et al., 2017). The diagnosis of mild TBI (mTBI) still lacks an interdisciplinary consensus regarding what constitutes an mTBI, and its determination has been largely epidemiological in nature. Since the majority of patients always obtained negative results from both CT and conventional MRI, there is still limited information about the neurobiological changes that are associated with clinical recovery.

Despite the negative findings in conventional imaging assessments, distinctive white matter tracts (WMTs), also known as diffuse axonal injury (DAI), after mTBI have been confirmed in both human biopsies (Bigler, 2010) and animal models (Mac Donald et al., 2007; Spain et al., 2010). Diffusion tensor imaging (DTI) has gained wide acceptance in clinical-research-based detection of abnormalities in mTBI patients (Basser and Pierpaoli, 1998; Arfanakis et al., 2002; Bigler, 2010). Water molecules in brain tissue are influenced by cellular structures and macromolecules by promoting anisotropy diffusion due to its various disparities in different orientations. Detection of water molecule diffusion fractional anisotropy (FA) can indicate the integrity of a cellular structure (Beaulieu, 2002). Although consistent observations indicate decreased anisotropic diffusion at more chronic periods post-injury, considerable debate remains on the direction (i.e., increased or decreased) of diffusion metrics on the acute to semi-acute phase (usually less than 2 months after injury) of mTBI (Arfanakis et al., 2002; Bazarian et al., 2007; Lipton et al., 2009; Chu et al., 2010; Mayer et al., 2010; Henry et al., 2011; Messé et al., 2012). Mayer et al. (2010) investigated 22 adult patients with mTBI within weeks post-injury and found increased FA noted in the genu, left superior coronal radiation (CR), and left uncinate fasciculus (UF), while decreased radial diffusivity (RD) was noted only in the genu, left CR, and left UF. Wilde et al. (2008) indicated the increased FA in the entire corpus callosum (CC) in a sample of 10 pediatric mTBI patients scanned between 1 and 6 days post-injury. Another study presented decreased FA in the splenium and posterior CC in 20 adult mTBI patients between 1 and 10 days post-injury (Inglese et al., 2005). Some studies have reported null findings during the semi-acute stages of mTBI. In a study, 12 adult mTBI patients were scanned within 3 months of injury (Rutgers et al., 2008). There were no significant differences between mTBI patients and controls in the CC.

The existing data on diffusion measurements in mTBI patients have key limitations regarding experimental design and analysis methods, motivating further investigation of injury patterns. Acute effects of injury, particularly edema, can confound such analysis. Longitudinal studies documenting serial changes in DTI metrics following mild TBI have yet to be performed (Meier et al., 2016; Churchill et al., 2017). One of the studies demonstrated that concussed athletes had significantly increased FA in the superior longitudinal fasciculus (SLF) than healthy athletes both at acute (within 1 week) and chronic periods (1 month). Further analyses indicated that this increase of FA in the SLF was driven by the decrease in RD rather than an increase in axial diffusivity (RAD; Meier et al., 2016). Another study found regions showing reduced average FA for athletes at acute concussion and at return-to-play relative to controls, including right corona radiata and bilaterally in posterior limbs of the internal capsule (Churchill et al., 2017). In addition, some researchers have enrolled small numbers of patients (e.g., <20) or had highly variable between-scan intervals, making the interpretation of between-subject variability difficult. Furthermore, most studies have lacked appropriate control groups for a longitudinal investigation (Meier et al., 2015). This is essential for modeling normal time-related changes in the brain measurements and neuropsychological ratings.

In the present study, DTI was collected to measure regional (i.e., voxel-wise) changes in FA values in the initial 7 days post-injury and the 1- and 3-month follow-up phase in a sample of mild TBI patients. To our knowledge, no studies have prospectively examined serial DTI changes during the normal course of recovery that typifies patients with mild TBI from these time course phase. To control the between-scan intervals, we confined this at a narrow interval. We mainly focused on the main time effect of the structural integrity of white matter (WM) fibers and its relationship with the initial neuropsychological measures. We hypothesized that the structural integrity loss in some tracts would normalize in patients with mild TBI as they transitioned from the acute to subacute injury phase and predicted that these deficits would relate to outcome measures, while other tracts will have persistent injury even beyond the first 3 months.

## Materials and Methods

### Participants

All consecutive patients with non-contrast head CT due to acute head trauma enrolled from the local ED formed the initial population. Inclusion criteria for all mild TBI patients were based on the World Health Organization’s Collaborating Centre for Neurotrauma Task Force (Borg et al., 2004): (i) Glasgow Coma Scale (GCS) score of 13–15 on presentation to the ED; (ii) one or more/any of the following: loss of consciousness (LOC) for less than 30 min, posttraumatic amnesia (PTA) for 24 h or less, and/or other transient neurological abnormalities such as focal signs, seizure, and intracranial lesion not requiring surgery; (iii) within 1 week after onset of a mild TBI (concussion); and (iv) age 16 years or older. Mild TBI patients were excluded for the following: (1) a history of a previous brain injury, neurological disease, long-standing psychiatric condition, or concurrent substance or alcohol abuse; (2) a structural abnormality on neuroimaging (CT and MRI); (3) intubation and/or presence of a skull fracture and administration of sedatives; (4) the manifestation of mild TBI due to medications by other injuries (e.g., systemic injuries, facial injuries, or spinal cord injury); (5) other problems (e.g., psychological trauma, language barrier, or coexisting medical conditions); and (6) TBI caused by penetrating craniocerebral injury. All of the patients were also screened for litigation to avoid bias in the assessment of neuropsychological tests (NPTs).

Healthy controls (HCs) with no history of neurological or psychiatric disorder were also recruited via the local imaging research facilities. Thirty-three patients with mTBI and 31 sex-, age-, and education-matched HC participated in the study. All participants were right-handed according to the Edinburgh Handedness Inventory (Oldfield, 1971).

### Standard Protocol

All the subjects gave written informed consent in person approved by a local institutional review board; the research procedures were approved by the Ethical Committee of the Second Affiliated Hospital of Wenzhou Medical University and conducted in accordance with the Declaration of Helsinki. MRI and neuropsychological assessments were performed on patients at both initial visit within 7 days post-injury (T1; median, 2 days; range, 0–5 days) and follow-up at both 1 month (T2; median, 37 days; range, 27–35 days) and 3 months (T3; median, 104 days; range 85–105 days). NPTs were performed within 48 h of MRI. The HCs received the first scanning within the same time range as the patients and follow-up 1 month (median, 35 days; range, 28–43 days) and 3 months (median, 108 days; range, 92–111 days) later. DTI and neuropsychological assessments were acquired in the full dataset of 33 patients and 31 HC at all three time points.

### Image Acquisition

A non-contrast CT scan was performed on all consecutive patients following acute head injury with a 64-row CT scanner (GE, Lightspeed VCT). The MRI scans were acquired with the use of a 3.0-T MRI scanner (GE 750). A custom-built head holder was used to prevent head movements. The MRI protocol involved the following: the high-resolution T1-weighted 3D MPRAGE sequence [echo time (TE) = 3.17 ms, repetition time (TR) = 8.15 ms, flip angle = 9°, slice thickness = 1 mm, field of view (FOV) = 256 mm × 256 mm, matrix size = 256 × 256], axial FLAIR (TR = 9,000 ms, TE = 95 ms, flip angle = 150°, thickness = 5 mm, slices = 20, FOV = 240 mm × 240 mm, matrix size = 173 × 256), axial susceptibility weighted imaging (TR = 37.8 ms, TE = 25 ms, flip angle = 15°, thickness = 2 mm, slices = 70, FOV = 230 mm × 230 mm, matrix size = 512 × 512), and diffusion-weighted imaging (TR = 7,300 ms, TE = 99 ms, flip angle = 90°, thickness = 3 mm, slices = 50, FOV = 256 mm × 256 mm, matrix size = 128 × 128, two averages, voxel size = 2 mm × 2 mm × 3 mm). DTI scan (*b* = 1,000 s/mm^2^) were acquired with 30 diffusion gradient orientations and the *b* = 0 repeated two times. The presence of focal lesions and cerebral microbleeds was independently determined by experienced clinical neuroradiologists (with 9 and 10 years’ experience) who assessed multiple modalities of neuroimaging data acquired at baseline [T1-flair, T2-flair, T2, susceptibility weighted imaging (SWI)]. Any disagreement between these two observers was resolved by consensus. None of the patients were with visible contusion lesions using conventional neuroimaging techniques or exhibited cerebral microbleeds on SWI.

### DTI Data Analysis

Preprocessing of the raw DTI data was performed using FSL software^1^. Motion and eddy current corrections were carried out by means of affine registration to the reference volume, and the corrected data were brain-extracted using FSL’s Brain Extraction Tool (BET). FA images were then created by fitting a tensor model to the raw diffusion data using the FMRIB Diffusion Toolbox (FDT). Voxel-wise statistical analysis of the FA data was performed using tract-based spatial statistics (TBSS), a tool included in the FSL software to examine WM diffusion in a whole-brain voxel-based manner. All the subjects’ diffusion metrics were subsequently aligned into a stereotactic coordinate system with the MNI152 template, using the FMRIB non-linear registration tool (FNIRT) with B-spline representation of the registration warp field. Next, all FA maps were averaged to produce a group mean FA image. The resultant mean FA map was then thinned to create a mean FA skeleton, representing the centers of all white-matter tracts in both study groups with a threshold FA value of 0.2. Statistical analysis was performed using the “randomize” command in FSL. The number of permutations was set to 10,000, and correction for multiple comparisons was achieved using threshold-free cluster enhancement (TFCE) with a family-wise error (FWE) rate of *P* < 0.05.

### Effect of White Matter Hyperintensities on Diffusion Tensor Imaging Analysis

Automated lesion segmentation was performed using the T1-weighted and T2-FLAIR image modalities on LST (Lesion Segmentation Tool) software package implemented in SPM12. LST determines gray matter (GM), WM, and cerebrospinal fluid (CSF) segmentations from T1-weighted images and computes the FLAIR intensity distributions of these tissue classes. The amount of “hyperintensity” of each voxel in terms of distance from the mean intensity of the WM, GM, and CSF distributions in the FLAIR image is crucial for defining a conservative lesion belief map (obtained by thresholding the GM belief map, initial threshold kappa was set to κ = 0.15) and a liberal lesion belief map (consisting of the sum of the three lesion belief maps). Lesion growing is then performed iteratively between the conservative and the liberal belief maps, until no more voxels are added to the lesions (Valverde et al., 2015). Finally, the subject-specific WM mask, generated by FAST in FSL (Zhang et al., 2001), was used to calculate the volume of WM hyperintensity based on the lesion belief map. Then, we tested whether the between-group differences were influenced by the individual WM hyperintensities.

### Neuropsychological Tests

Comprehensive NPTs were assessed: (i) Trail-making test part A (TMT-A) (Arnett and Labovitz, 1995) and WAIS-III Digit Symbol Coding score (DSC) (Wechsler et al., 1997) to examine cognitive information processing speed; (ii) forward digit span (FDS) and backward digit span (BDS) of the Wechsler Adult Intelligence Scale WAIS-III (Harman-Smith et al., 2013) to assess working memory; and (iii) verbal fluency test (VFT; Troyer et al., 1997) to assess verbal fluency including language ability, semantic memory, and executive function. Self-reported symptomatology included the following: the Rivermead post-concussion symptoms questionnaire (RPCS; King et al., 1995), Posttraumatic stress disorder Checklist – Civilian version (PCL-C) (Bliese et al., 2008), Beck Depression Inventory 2nd edition BDI-II (beck; Beck et al., 1996), Fatigue Severity Scale (FSS; Krupp et al., 1989), and Insomnia Severity Index (ISI; Bastien et al., 2001). We have conducted the “reliable change indices” analysis for all of NPTs in the present study. This method provides an estimate of the probability that a given difference score would not be obtained by chance; that is, the score would not be due to measurement error (Iverson, 2001; Baxendale and Thompson, 2005). The standard error of difference (*S*_diff_) provides the clinician with an estimate of possible measurement error relating to test–retest scores.

### Statistical Analyses

SPSS 19.0 software was used (IBM Corp., Armonk, NY, United States). The Shapiro–Wilk *W*-test was used to test for normality distribution of all continuous variables. The independent two-sample *t*-test and the Mann–Whitney test were used to compare group differences based on data normality, respectively. Chi-square analyses were applied to assess categorical variables. Effect sizes (Cohen’s *d*) were computed to demonstrate the magnitude of observed differences. Longitudinal analysis, via repeated-measures ANOVA, was conducted to examine changes in both neuropsychological assessments and imaging FA metric as a function of recovery. This analysis only included the group and time as terms with no further control variables. Spearman’s correlation coefficient was used to examine the association between FA metric of WMTs and neuropsychology test scores. The significance level was adjusted by using the Bonferroni correction with *P* < 0.05. None of the patients were with visible contusion lesions using neuroimaging techniques. Three patients exhibited cerebral microbleeds on SWI. All of the following analyses were also conducted in patients without microbleeds to investigate the influence of the presence of microbleeds on final results. We have also conducted the between-group difference in the TBSS analysis and used the individual WM hyperintensities observed in the T2-FLAIR sequence as regressors.

## Results

### Demographic and Initial Neuropsychological Data

There were no significant differences in the age (Cohen’s *d* = 0.01, *P* = 0.956), years of education (Cohen’s *d* = -0.49, *P* = 0.083), or gender (Cohen’s *d* = 0.16, *P* = 0.36) between the mTBI and control groups. The mTBI patients’ NPT differed significantly from controls on a number of tests at initial admission (RPCS, PCL-C, beck, and ISI, all for Cohen’s *d* > 0.9 and *P* < 0.005); however, some tests had no significant difference (TMT-A, DSC, FDS, BDS, VFT, and FSS all for *P* > 0.05). All demographic and clinical characteristics for patients with mild TBI and HC were presented in Table 1.

**Table 1:** Demographic and neuropsychological data for mTBI and control participants.

	mTBI mean (SD)	Controls mean (SD)	mTBI vs. controls *P*-value (Cohen’s *d*)
**Demographic**			
Age	37.7 (13.6)	37.5 (12.2)	0.96 (0.01)
Gender (M/F)	21/12	22/9	0.36 (0.16)
Education	8.1 (4.1)	10.5 (5.9)	0.083 (-0.49)
**Neuropsychology**			
TMT-A	64.1 (31.7)	55.1 (37.0)	0.30 (0.26)
RPCS	11.0 (7.3)	2.8 (2.7)	< 0.001 (1.48)
PCL-C	23.9 (6.0)	17.0 (0.0)	< 0.001 (1.64)
DCS	31.8 (15.0)	42.7 (17.6)	0.010 (-0.67)
FDS	7.6 (1.7)	8.0 (1.5)	0.34 (-0.24)
BDS	3.6 (1.4)	3.8 (1.3)	0.56 (-0.14)
VFT	17.3 (5.1)	18.0 (6.7)	0.62 (-0.12)
Beck	4.4 (4.7)	0.1 (0.2)	< 0.001 (1.31)
FSS	10.4 (4.2)	9.0 (0.0)	0.07 (0.48)
ISI	7.3 (6.0)	2.7 (3.7)	0.001 (0.92)

*mTBI, mild traumatic brain injury; TMT-A, Trail-making test part A; RPCS, Rivermead post-concussion symptoms questionnaire; PCL-C, Posttraumatic Stress Disorder checklist – Civilian version; DSC, Wechsler Adult Intelligence Scale WAIS-III Digit Symbol Coding score; FDS and BDS, forward digit span and backward digit span of the Wechsler Adult Intelligence Scale WAIS-III; VFT, Verbal fluency test; Beck, Beck Depression Inventory 2nd edition BDI-II; FSS, Fatigue Severity Scale; ISI, Insomnia Severity Index. The results are thresholded at *P* < 0.05*.

### Neuropsychological Performance and Changes Over Time

Our results show that the majority of NPTs were sorted to the “most reliable” group, with the HCs having the most reliable change indices, given the relatively small practice effects (Supplementary Figure S1). However, only the Trail-making Test A presented the practice effects across the three time points. Thus, we were more cautious in explaining the corresponding changes in the Trail-making Test A in patients. For the tests with reliable change indices, the percentages of patients who demonstrated a significant improvement or decline were illustrated in Supplemetary Figure S2. For the DCS, about one-third of patients deteriorated and another one-third of patients improved between initial acute phase (T1) and follow-up 1 month post-injury (T2). For the FDS and BDS, one-third of patients presented improvement between T1 and T2. For the VFT, 20% of patients deteriorated and 15% of patients improved between T1 and T2.

Longitudinal analyses were first conducted to examine changes in the NPT as a function of recovery. A main effect of time was only observed in the DSC [*F*(2,60) = 4.14, *P* = 0.018]. For the DSC, scores were significantly improved at T2 and T3 relative to T1 (*P* = 0.003 and *P* < 0.001) and differences became non-significant at T2 and T3 following multiple comparison correction (*P* > 0.1). The main effect of group was significant for the RPCS, PCL-C, and beck (all for *P* < 0.05). Scores on the RPCS, PCL-C, and beck were significantly higher than HCs at T1 (*P* < 0.001), T2 (*P* < 0.005), and T3 (*P* < 0.002) following multiple comparison correction.

### Diffusion Metrics Differences and Recovery

**Figure 1:**
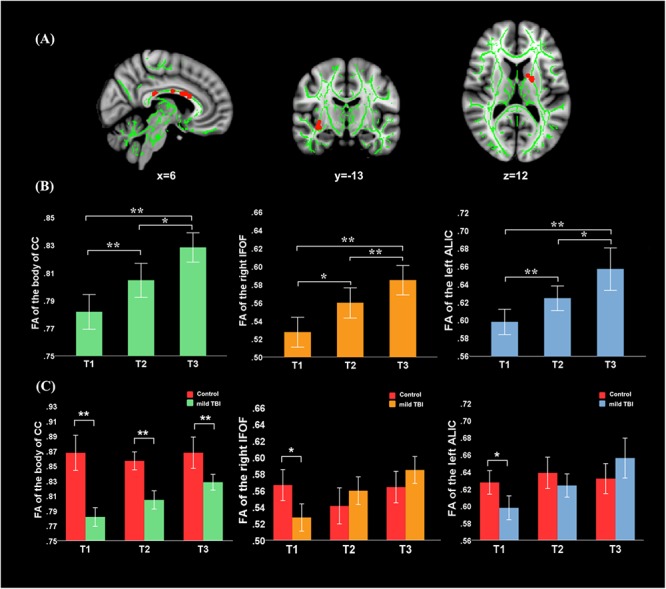
Main effect of time and *post hoc* results in the fractional anisotropy (FA). **(A)** FA showed significant main effect of time in the body of CC, left anterior limb of internal capsule (ALIC), and right inferior fronto-occipital fasciculus (IFOF). **(B)** For these three tracts, *post hoc* analyses with Bonferroni correction revealed that FA values were significantly lower at T1 (*P* < 0.001) and T2 (*P* < 0.005) relative to the T3, and presented salient difference between T1 and T2 (*P* < 0.005). **(C)** Significant differences in the left ALIC and right IFOF FA values in patients relative to controls only at T1 (*P* < 0.005) but returned to the normal level at both T2 (*P* > 0.1 for all) and T3 (*P* > 0.08 for all). The body of CC exhibited the continuing loss of integrity at T1, T2, and T3 (*P* < 0.001) compared to healthy controls (HCs). Error bars represent 95% confidence intervals. ^∗^Significant Bonferroni corrected for multiple comparisons (^∗^*P* < 0.005; ^∗∗^*P* < 0.001).

There was no significant interaction effect (group × time) for the WMTs. A significant main effect of time was located in the left anterior limb of internal capsule (ALIC) [*F*(1,61) = 9.49; *P* = 0.0023], right inferior fronto-occipital fasciculus (IFOF) [*F*(1,61) = 9.33; *P* = 0.0025], and body of CC [*F*(1,61) = 8.94; *P* = 0.0031] (Figure 1). For these three tracts, *post hoc* analyses with Bonferroni correction revealed that FA values were significantly lower at T1 (*P* < 0.001) and T2 (*P* < 0.005) relative to T3 and presented salient difference between T1 and T2 (*P* < 0.01). Mild TBI data from the left ALIC, right IFOF, and body of CC were next compared with those of HCs using *post hoc* analyses with Bonferroni correction. We found significant differences in the left ALIC and right IFOF FA values in patients relative to controls only at T1 (*P* < 0.005) but presented no significant difference at both T2 (*P* > 0.1 for all) and T3 (*P* > 0.08 for all) compared with HCs. The body of CC exhibited persistent loss of integrity at T1, T2, and T3 (*P* < 0.001) compared to HCs.

**Figure 2:**
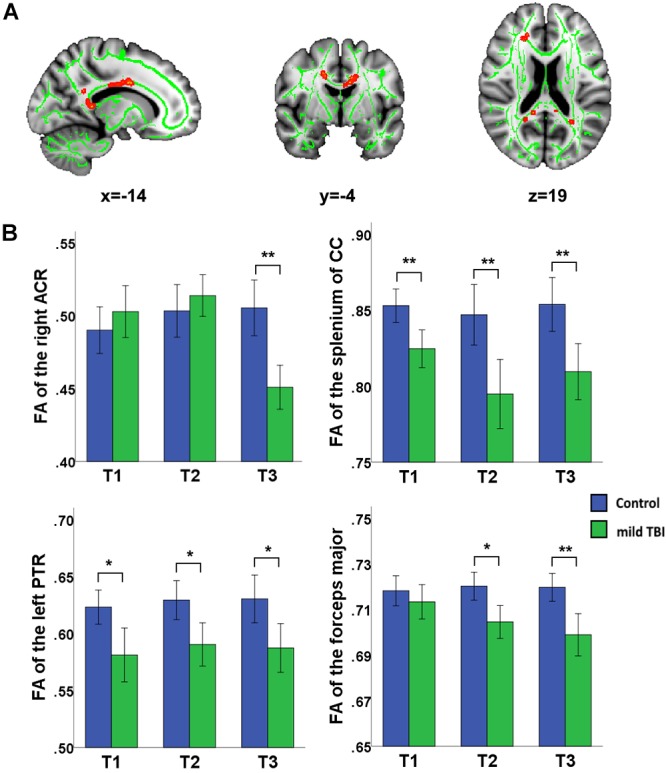
Main effect of group and *post hoc* results in recovery of white matter tracts (WMTs). **(A)** Significant effect of group was presented in the right ACR, left posterior thalamic radiation (PTR), forceps major, body of corpus callosum (CC), and splenium of CC. **(B)** *Post hoc* analyses with Bonferroni correction further revealed that FA values in the left PTR, body of CC, and splenium of CC were reduced at T1, T2, and T3 relative to the HCs (*P* < 0.005 for all). The right ACR presents only loss of integrity at the later phase T3 rather than T1 and T2 (*P* < 0.001). For the forceps major, the reduced FA only presented at later phase at T2 (*P* = 0.0012) and T3 (*P* < 0.001) but not at T1. ^∗^Significant Bonferroni corrected for multiple comparisons (^∗^*P* < 0.005; ^∗∗^*P* < 0.001).

Significant effect of group was presented in the right anterior corona radiata (ACR), left posterior thalamic radiation (PTR), forceps major, body of CC, and splenium of CC (Figure 2). *Post hoc* analyses with Bonferroni correction further revealed that FA values in the left PTR, body of CC, and splenium of CC were reduced at T1, T2, and T3 relative to the HCs (*P* < 0.005 for all). The right ACR presented loss of integrity only at T3 (*P* < 0.001) and not at T1 and T2. For the forceps major, the reduced FA only presented at T2 (*P* = 0.0012) and T3 (*P* < 0.001) and not at T1. The combination of multiple parameters (MD, RD, and AD) from DTI was very helpful to understand the possible physiopathology mechanism following mild TBI. We also supplemented other diffusion metrics that presented reduced FA in mild TBI patients, compared with controls (Supplementary Table S1). Our findings showed that most between-group differences were not influenced by the individual WM hyperintensities. Only the between-group difference in the left PTR at 1 month post-injury following mild TBI disappeared after regressing for the WM hyperintensities (Supplementary Table S2).

### Relationship Between Outcome and Other Variables

**Figure 3:**
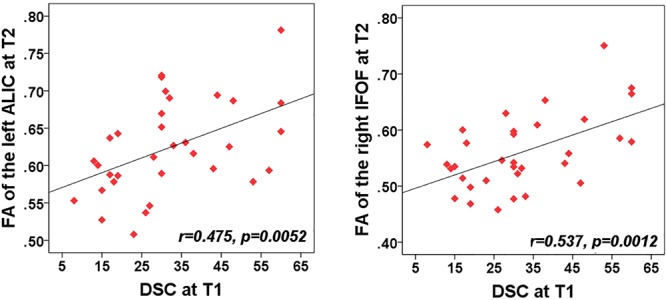
Correlation between the FA of WMTs with clinical outcomes. Patient’s cognitive information processing speed performance (rated by digit symbol coding, DSC) at T1 was positively related with FA value of the left anterior limb of internal capsule (ALIC) at T2 (*r* = 0.48, *P* < 0.005). Correlations of the DSC at T1 and FA value in the right IFOF at T2 (*r* = 0.5, *P* = 0.003) also showed significant positive values after Bonferroni correction.

The purpose of correlation analysis focused on the assessment of the relationship between the altered integrity of WMTs and neuropsychological performances within each time point and across time points. We were mainly concerned about both neuropsychological testing and WMTs showing the main effect of time. For the NPTs, a main effect of time was only observed in the DSC [*F*(2,60) = 4.14, *P* = 0.018]. For the fibers, a significant main effect of time was located only in the left ALIC [*F*(1,61) = 9.49; *P* = 0.0023], right IFOF [*F*(1,61) = 9.33; *P* = 0.0025], and body of CC [*F*(1,61) = 8.94; *P* = 0.0031]. The following analysis was conducted. Firstly, a correlation analysis between the clinical measure (DSC) and these three tracts for each time point was carried out. The FA value of these three tracts presented no significant associations with the DSC at any specific time points (all for *P* > 0.2). Additional analysis found that there was no significant correlation between altered DSC scores and FA values between T1 and T2, between T1 and T3, and between T2 and T3 in these three fibers (all for *P* > 0.2). Finally, considering that the left ALIC and right IFOF showed significant between-group differences at initial assessment but showed no significant difference at T2 and T3, we wanted to explore whether there was any association of the baseline DSC performance with the WM recovery in these two tracts. Exploratory analyses (Bonferroni correction at *P* < 0.05) were thus performed to assess the relationship between T1 clinical measure (DSC) with the left ALIC and right IFOF tracts at both T2 and T3, respectively. Results indicated that initial DSC performance can predict better recovery in the structural integrity of the left ALIC (*r* = 0.48, *P* = 0.005) and right IFOF (*r* = 0.54, *P* = 0.0012) at T2 after Bonferroni correction (*P* < 0.05/2, Figure 3). These findings may infer that an individual patient with better performance on the DSC at the acute phase can obtain better recovery in these two WMTs 1 month post-injury following mild TBI.

**Figure 4:**
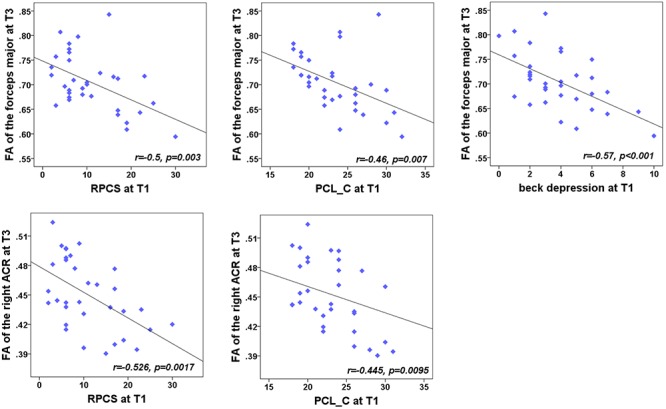
Correlation between the FA of WMTs with clinical outcomes. At T3, the results indicated a negative relationship of the right anterior corona radiata (ACR) with both the post-concussion severity (rated by RPCS, *r* = -0.48, *P* < 0.005) and posttraumatic stress (PCL-C; *r* = -0.49, *P* = 0.0037). Correlations with the forceps major and RPCS, PCL-C, and Beck depression (*r* = -0.5, *P* = 0.003; *r* = -0.49, *P* = 0.0037; *r* = -0.57, *P* < 0.001) also showed significant negative values after Bonferroni correction.

We were also concerned with both neuropsychological testing and WMTs showing the main effect of group. For the NPTs, a main effect of time was only observed in the RPCS, PCL-C, and Beck depression (all for *P* < 0.05). For the fibers, a significant main effect of time was only located in the right ACR, left PTR, forceps major, body of CC, and splenium of CC. For each time point, correlation analyses showed that the FA value of these five tracts presented no significant correlations with these syndromes at any specific time point (*P* > 0.05). We also want to explore whether the baseline assessments of these NPTs were associated with the later integrity of WMTs at T2 and T3 follow-up phases. Results showed that the significance only existed at T3 (Figure 4). Results indicated a negative relationship of the right ACR with both baseline post-concussion severity (rated by RPCS, *r* = -0.53, *P* = 0.0017) and posttraumatic stress (PCL-C) (*r* = -0.45, *P* = 0.0095) but did not survive correction for multiple comparisons (*P* < 0.05/3 × 5). Correlations of the forceps major with RPCS and Beck depression (*r* = -0.5, *P* = 0.003; *r* = -0.57, *P* < 0.001) also showed significant negative values after Bonferroni correction. The loss of structural integrity in the forceps major also presented more complaints in the baseline PCL-C but did not survive correction for multiple comparisons (*r* = -0.46, *P* = 0.007). These findings inferred that patients with more complaints on the post-concussion syndromes at baseline have more loss of structural integrity in the right ACR and forceps major. Other correlations between baseline syndromes and later WM injury did not obtain significance.

## Discussion

To our knowledge, this is the first study to investigate longitudinal changes in WMTs from the acute through subacute phases to 3 months post-injury following mTBI. In a homogeneous sample of mTBI, we found decreased FA values in the left ALIC and right IFOF during the 7 days post-injury, which showed longitudinal evidence of recovery following 1 month post-injury. Increased FA values in these two tracts at 1 month post-injury were positively associated with better performance on cognitive information processing speed at initial assessment. By contrast, there were also some tracts (right ACR, forceps major, and body of CC) exhibiting the persistent loss of integrity sustaining even beyond 3 months, which can also predict the persisting post-concussion syndromes, posttraumatic stress, and depression even beyond 3 months post-injury. Crucially, the persistent loss of structural integrity in these tracts may contribute to the persistent post-concussion syndromes in mild TBI patients.

Diffusion tensor imaging is widely used *in vivo* studies on WM injury of TBI (Shenton et al., 2012). Among severe and chronic TBI patients, DTI scans can determine the altered FA values in fiber tracts, wherein the mechanism had been illustrated in previous studies with animal models or humans and was considered as the result of diffuse degenerative changes such as Wallerian degeneration, axonal collapse, and myelin degeneration (Albensi et al., 2000; Arfanakis et al., 2002; Inglese et al., 2005; Li et al., 2011). However, due to the preliminary nature of these studies, there is still controversy regarding the DTI metric changes in mild TBI patients. Indeed, there are several differences regarding the pathological changes between chronic and acute and subacute TBI patients (Povlishock and Katz, 2005; Newcombe et al., 2007). The cellular injury in the acute phase is volatile and may, in cases, stay the same, ameliorate gradually, or deteriorate; thus, it is thought to be a very dynamic process (Albensi et al., 2000; Povlishock and Katz, 2005). In addition to this characteristic of axon injury during the acute and subacute phases, the injuries of mTBI patients also differ from those of moderate to severe TBI patients. Until now, it is still unclear how these different neuropathological changes (individually or combined) affect the DTI test results. Indeed, many studies have reported differences in axial diffusivity and have been inconsistent, with some groups finding increases (Sidaros et al., 2008; Tasker et al., 2010), decreases (Henry et al., 2011), and no change (Mac Donald et al., 2007), and some regions showed axial diffusivity increases while others showed decreases (Farbota et al., 2012). Therefore, it is premature to suggest a physiopathology mechanism based on imaging data alone. This is a limitation for our study as well as most other current studies. These pathological changes occur at different points of time and have different effects on axons and neurons, which may explain the variable results of the FA values. To overcome these variabilities, all of the participants in the present study were evaluated using both neurological and imaging assessments at a narrow time interval.

The acute phase is thought to be a very dynamic process, and previous studies present bidirectional changes in FA of mTBI patients (Sidaros et al., 2008; Tasker et al., 2010; Henry et al., 2011). Histopathological studies with animal and human tissues show the acute stage of mTBI, and the pathological change of axon microstructure is a dynamic process with multiple variable effects. These inconsistencies may be partly due to the highly variable post-injury days for acute phase, such as 1–6 days (Chu et al., 2010; Yallampalli et al., 2013), within 72 h (Bazarian et al., 2007; Toth et al., 2013), and within 24 h (Arfanakis et al., 2002), or limited samples sizes, such as 5 patients in Arfanakis’s study (Arfanakis et al., 2002), 6 in Bazarian’s study (Bazarian et al., 2007), 10 in Chu’s study (Chu et al., 2010), and 11 in Yallampalli’s study (Yallampalli et al., 2013), making the interpretation of between-subject variability difficult. In the present study, decreased FA values were observed in the left ALIC and right IFOF within acute 7 days post-injury and no bidirectional changes were detected. The result presented in this report highlights the need of strictly standardized image acquisition time points for mTBI patients.

For mTBI injuries, partial pathological changes are usually transient, but may persist in some cases. The pathological changes at 1 month post-injury are, however, not the research focus in previous studies. We found that FA values of the right left ALIC and right IFOF in patients were firstly reduced at the initial acute phase but presented no significant difference after 1 month post-injury. Previously, Toth et al. (2013) found that the mTBI patients showed decreased FA values in nearly all the WMTs 72 h after injury; however, after 1 month of injury, the FA values remained decreased in some tracts. Similarly, in a homogeneous sample of collegiate athletes with sports-related mTBI, Meier et al. (2015) observed decreased CBF in the right dorsal middle insula cortex and superior temporal sulcus during the first week post-injury, which showed recovery at 1 month post-injury. Additionally, in athletes with concussion, Vagnozzi et al. (2010) demonstrated that a reduction in the ratios of *N*-acetylaspartate to creatine and choline is pronounced at 3 days post-concussion but fully recovered by 1 month. The recovery of FA could reflect reorganization within the WM, due to axonal recovery or even regrowth (Sidaros et al., 2008). We found some support for this proposal by specifically examining the left ALIC and right IFOF, where the increased FA values at 1 month post-injury were positively associated with better performance on cognitive information processing speed at initial assessment. The ALIC connects the thalamus and prefrontal cortex, which conveys cognitive and corticothalamic fibers, and the right IFOF connects occipital, temporal, and frontal lobes, which has an important role in reading, attention, visual perception, processing and memory, and language; thus, such connections likely influence higher-order cognition (Martino et al., 2010). This result therefore provides support for the performance that 1 month post-injury is an important phase when the normalization of axial diffusivity in some WM occurs. Although statistical unification of neuroimaging data obtained acutely and subacutely (e.g., 1 month) after injury does not seem justifiable, these findings suggest that the MRI-detectable abnormality developing in the acute phase resolves dynamically in the first month after mild injury. Therefore, to determine whether the injuries obtained by complex and variable pathogenic mechanisms during the early stage might persist throughout the later stages of disease, 1 month post-injury was rather important.

By contrast, there were also some tracts (right ACR, forceps major, and body of CC) exhibiting the persistent loss of integrity sustaining beyond 3 months. Crucially, the correlations between FA and improved cognitive deficits at 3 months were positive: higher FA in right ACR and forceps major was associated with better outcomes. ACR connects to the internal capsule and forceps major connects to prefrontal and fronto-orbital regions, which may be related to perceptual and cognitive functions. Symptoms such as dizziness, fatigue, poor concentration, and memory problems experienced by chronic mTBI patients are thought to result from these lesions (Messé et al., 2011; Khong et al., 2016). Hence, the persistent loss of structural integrity in these tracts may contribute to the persistent post-concussion syndromes in mTBI patients, which can also predict the persisting post-concussion syndromes, posttraumatic stress, and depression even beyond 3 months post-injury. The identification of such predictive tracts (right ACR and forceps major) may help to better stratify patients early and to refine the concept of mTBI severity beyond traditional symptoms of alteration.

There were some limitations in the current study. Voxel-based analyses (i.e., TBSS) included the alignment of WMTs. In fact, such voxel-wise analysis is based on the assumption that clinically heterogeneous patients have a homogeneous (i.e., high degree of spatial overlap) spatial overlap pattern of WM abnormalities limited to detect the relative subtle diffuse “signals” influenced by the heterogeneity in injury location of mild TBI. Some studies adopted a new approach for quantifying diffusion abnormalities through a metric similar to lesion load (White et al., 2009; Ling et al., 2012). Specifically, clusters of abnormally high or low anisotropic diffusion were determined on a voxel-wise basis and then summed to represent total burden of distributed pathology. Further studies are needed to capture the individual-based “fine” diffuse patterns of WM abnormalities.

## Conclusion

In conclusion, this study characterizes the dynamic variation rules of WM fibers within the first 3 months following mTBI. The results indicated that the pathological changes of fiber tracts at the acute phase were unstable and subject to a dynamic changing process. Therefore, we suggest a strict homogeneity of samples, and strictly standardized image acquisition time points for mTBI patients are required in subsequent studies. We found that FA values of the right left ALIC and right IFOF in patients were firstly reduced at the initial acute phase but presented no significant difference after 1 month post-injury compared with HCs. Hence, we suggest that 1 month post-injury is an important phase to determine whether the injuries obtained during the acute stage might persist throughout the chronic stages of mTBI. Finally, we also suggest that decreased FA values in right ACR and forceps major may help distinguish patients with posttraumatic stress disorder (PTSD) from mTBI patients at the acute and subacute stage.

## Data Availability

All datasets generated for this study are included in the manuscript and/or the Supplementary Files.

## Ethics Statement

All the subjects gave written, informed consent in person approved by a local institutional review board; the research procedures were approved by the Ethical Committee of The Second Affiliated Hospital of Wenzhou Medical University and conducted in accordance with the Declaration of Helsinki.

## Author Contributions

BY and MZ contributed to the conception and design of the study. D-DL and HH organized the database. L-XH performed the statistical analysis. D-DL and C-HG wrote the first draft of the manuscript. J-FZ and G-HB wrote the sections of the manuscript. All authors contributed to the manuscript revision, read, and approved the submitted version.

## Conflict of Interest Statement

The authors declare that the research was conducted in the absence of any commercial or financial relationships that could be construed as a potential conflict of interest.

## Footnotes

1 http://www.fmrib.ox.ac.uk/fsl/index.html
