# Supplementary material for: Longitudinal Changes in Diffusion Tensor Imaging Following Mild Traumatic Brain Injury and Correlation With Outcome
Source: Front Neural Circuits. 2019 May 7;13:28. doi: 10.3389/fncir.2019.00028 (PMC6514143; doi:10.3389/fncir.2019.00028)
Supplement: Supplementary file 1 [file Table_1.docx]

Supplementary Table S1. Diffusion metrics changes following both initial and follow-up post-injury of mild TBI (red means the increased diffusion metrics in patients compared controls).

| main effect of group | T1 | T2 | T3 |
| --- | --- | --- | --- |
| **MD** |  |  |  |
| right ACR | F(1,62)=3.753, P=0.057 | **F(1,62)=4.360, P=0.041** | **F(1,62)=7.090, P=0.01** |
| body of CC | **F(1,62)=4.205, P=0.045** | **F(1,62)=5.970, P=0.017** | **F(1,62)=5.987, P=0.017** |
| forceps major | F(1,62)=2.902, P=0.093 | F(1,62)=1.710, P=0.196 | F(1,62)=2.574, P=0.114 |
| left PTR | F(1,62)=2.698, P=0.106 | F(1,62)=1.124, P=0.293 | F(1,62)=2.450, P=0.123 |
| splenium of CC | **F(1,62)=12.353, P=0.001** | **F(1,62)=16.090, P<0.001** | **F(1,62)=14.762, P<0.001** |
| **AD** |  |  |  |
| right ACR | F(1,62)=0.345, P=0.559 | F(1,62)=0.001, P=0.984 | F(1,62)=0.110, P=0.741 |
| body of CC | F(1,62)=2.509, P=0.118 | F(1,62)=3.243, P=0.077 | F(1,62)=0.727, P=0.397 |
| forceps major | F(1,62)=0.807, P=0.373 | F(1,62)=2.353, P=0.130 | F(1,62)=2.011, P=0.161 |
| left PTR | F(1,62)=0.035, P=0.853 | F(1,62)=1.592, P=0.212 | F(1,62)=0.062, P=0.805 |
| splenium of CC | F(1,62)=0.012, P=0.914 | F(1,62)=0.354, P=0.554 | F(1,62)=0.197, P=0.659 |
| **RAD** |  |  |  |
| right ACR | **F(1,62)=5.220, P=0.026** | F(1,62)=3.871, P=0.054 | **F(1,62)=6.837, P=0.011** |
| body of CC | **F(1,62)=7.684, P=0.007** | **F(1,62)=13.694, P<0.001** | **F(1,62)=10.308, P=0.002** |
| forceps major | **F(1,62)=11.147, P=0.001** | **F(1,62)=10.413, P=0.002** | **F(1,62)=9.306, P=0.003** |
| left PTR | **F(1,62)=4.899, P=0.031** | **F(1,62)=4.982, P=0.029** | **F(1,62)=5.233, P=0.026** |
| splenium of CC | **F(1,62)=23.628, P<0.001** | **F(1,62)=31.536, P<0.001** | **F(1,62)=27.026, P<0.001** |
