# Supplementary material for: Longitudinal Changes in Diffusion Tensor Imaging Following Mild Traumatic Brain Injury and Correlation With Outcome
Source: Front Neural Circuits. 2019 May 7;13:28. doi: 10.3389/fncir.2019.00028 (PMC6514143; doi:10.3389/fncir.2019.00028)
Supplement: Supplementary file 2 [file Table_2.docx]

**Supplementary Table S2.** The between-group differences after regressed for the individual white matter hyperintensity.

| Fiber Tracts | T1 | T2 | T3 |
| --- | --- | --- | --- |
| left ALIC | P = 0.002 | P = 0.09 | P = 0.434 |
| right IFOF | P = 0.024 | P = 0.543 | P = 0.436 |
| body of CC | P < 0.001 | P < 0.001 | P < 0.001 |
| right ACR | P = 0.663 | P = 0.392 | P < 0.001 |
| left PTR | P = 0.039 | P = 0.060 | P = 0.037 |
| Splenium of CC | P = 0.014 | P = 0.042 | P < 0.001 |
| forceps major | P = 0.868 | P = 0.003 | P < 0.001 |

Abbreviations: ALIC, anterior limb of internal capsule; IFOF, inferior fronto-occipital fasciculus; CC, corpus callosum; ACR, anterior corona radiate; PTR, posterior thalamic radiation; 7 days post-injury (T1, median 2 days, range 0-5 days) and follow-up at both 1 month (T2, median 37 days, range 27-35 days) and 3 months (T3, median 104, range 85-105 days).
