# Supplementary figures and images for: Longitudinal Changes in Diffusion Tensor Imaging Following Mild Traumatic Brain Injury and Correlation With Outcome
Source: Front Neural Circuits. 2019 May 7;13:28. doi: 10.3389/fncir.2019.00028 (PMC6514143; doi:10.3389/fncir.2019.00028)

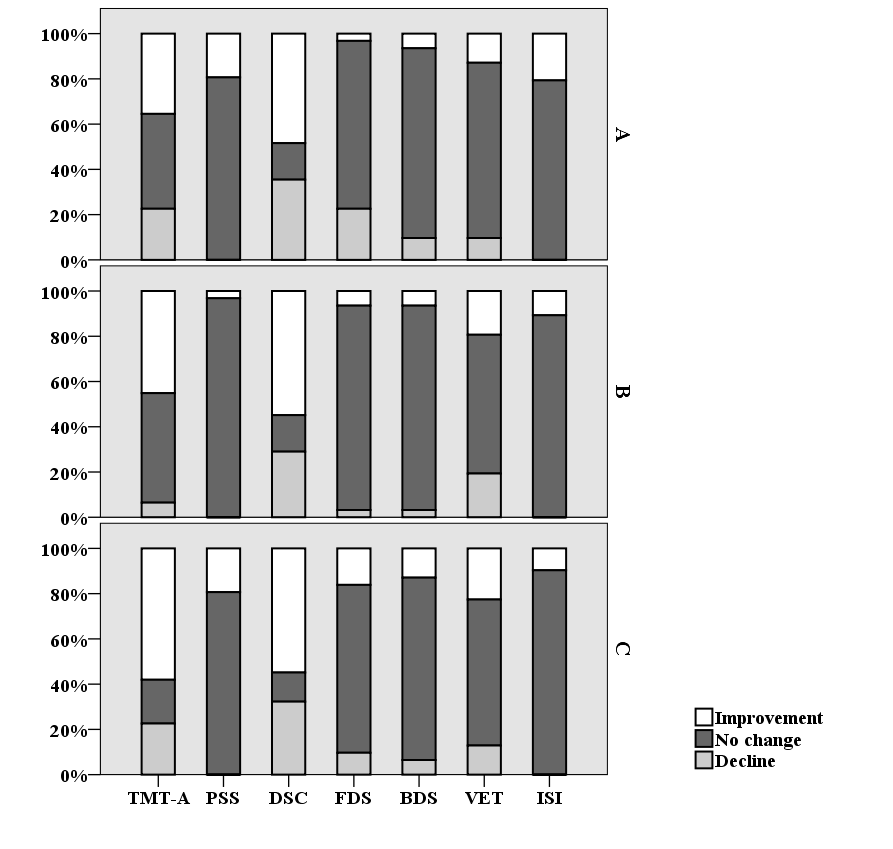

Supplement: Supplementary file 3 [file Image_1.tif]

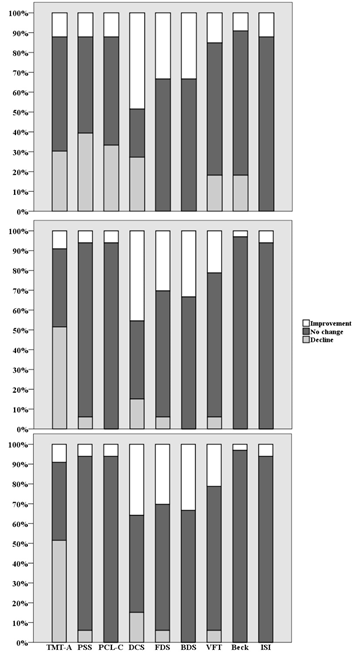

Supplement: Supplementary file 4 [file Image_2.tif]
